# Language is the missing link in action-perception coupling: an EEG study

**DOI:** 10.1038/s41598-020-71575-w

**Published:** 2020-09-03

**Authors:** Pauline Billard, Sélim Yahia Coll, Donald Glowinski, Didier Grandjean

**Affiliations:** Neuroscience of Emotion and Affective Dynamics Laboratory, Faculty of Psychology and Educational Sciences, Swiss Centre for Affective Sciences, University of Geneva, 1205 Geneva, Switzerland

**Keywords:** Neuroscience, Psychology

## Abstract

The paper reports an electrophysiological (EEG) study investigating how language is involved in perception–action relations in musically trained and untrained participants. Using an original backward priming paradigm, participants were exposed to muted point-light videos of violinists performing piano or forte nuances followed by a congruent vs. incongruent word. After the video presentation, participants were asked to decide whether the musician was playing a piano or forte musical nuance. EEG results showed a greater P200 event-related potential for trained participants at the occipital site, and a greater N400 effect for untrained participants at the central site. Musically untrained participants were more accurate when the word was semantically congruent with the gesture than when it was incongruent. Overall, language seems to influence the performance of untrained participants, for which perception–action couplings are less automatized.

## Introduction

Humans are experts in decoding each others' gestures allowing them to respond efficiently to the multitude of unpredicted situations they are facing^1^. However, in daily-life situations, gestures can be misinterpreted resulting in a poor capacity to react. For instance, in a joint action such as moving a sofa with a partner, one would nod to indicate the time to release the weight but the partner may interpret differently and will move forward, putting the first at risk to let the sofa down and break it. In specific contexts such as sport and music, motor expertise can contribute to a better coordination as gestures require specific technics and relate to a set of extensively learnt actions shared among experts. In music and sport, the ability to predict the dynamic of a gesture is crucial due to both time constraints and the requirement of a precise gesture in a joint action (e.g., throwing a ball in a basket, playing a specific nuance on an instrument^2^). In musical context, action-perception coupling and their related predictions are highlighted in interference paradigm where musically trained (i.e. musicians) and untrained (i.e. non musicians) participants are asked to produce a specific instrument-relevant gesture (e.g., play a chord on a piano) in response to a visual and/or auditory stimulus (e.g., a specific chord accompanied with its sound) either congruent or incongruent with the perceptual feedback expected^3^. These studies showed that musicians are slower to produce the gesture when the percept and movement are incongruent (e.g., the sound of the chord played does not match the sound of the imperative chord), but this effect has not been obtained with non-musicians^3,4^. Musicians’ sensorimotor system properties is enriched by extensive training and execution of specific music-related movements. These properties might form the basis of an “internal (forward) model”^5–7^, hypothesizing that the representations of a motor action are reactivated during observation and execution of the same or related action^8^. Thus, motor expertise gained via instrument-specific training could be linked with perceptual representations reactivated when the action is about to be made, which might help the coordination in increasing the synchrony between performers^9^.

If a growing body of evidence confirms that motor training supports action understanding, the role of language has also been emphasized in this process^10–12^. Actually, numerous works have shown a co-dependency between action and language^13^. Language seems to boost action perception^14^ and motor training to improve action-related language understanding^15^. Studies using functional imaging and transcranial magnetic stimulation have highlighted the implication of brain regions primarily involved in action recognition and control in linguistic processing tasks^16–18^. The impact of language on action understanding has mainly been investigated at the semantic level^19–21^. Bernardis and Caramelli^20^ developed a priming paradigm, combining words that primed the presentation of iconic gestures (i.e. gestures conveying direct meaning) in two experimental conditions: Congruent (e.g., the word guitar followed by the pantomime of an actor playing guitar) and incongruent (e.g., the word walk followed by the pantomime of an actor playing guitar). They found a facilitation effect on the gesture recognition when the word and the iconic gesture were congruent and an interference effect in the opposite condition. According to the authors, the gesture and linguistic meaning systems work jointly in daily-life communication to shape the meaning characteristics of social interactions. This view is in accordance with McNeill’s model^22^ postulating that gesture and speech are tightly integrated, each providing specific and redundant information unified in an enriched representation. The interference effect is taken as evidence of this integration: In the incongruent condition, meaning of both language and gesture would compete with each other revealing a semantic interaction (i.e. the meaning system trying to combine gesture and speech^23^). Language and action are tightly related but whether their interaction is influenced by expertise, (i.e. in the situation where action-perception coupling is largerly automatized) is not yet known. A musical context seems to be an appropriate experimental framework to answer such question. Several studies have used musical extracts to investigate congruency detection in semantically related or unrelated context^24,25^. However, the effect of the underlying gestures enacted during the music performance remains unexplored. In addition, using a musical context instead of the usual iconic gestures^19–21^ allows us to investigate a wider range of gesture variations, more representative of gesture’s complexity observed in daily life settings. It also provides the advantage of remaining in a well-delimited environment (i.e. gesture related to the field of music performance) suitable for experimental research. Whereas observers uniformly recognize iconic gestures, musical gestures are perceived differently according to the performance itself and according to the oberserver’s level of expertise. In sum, musical gestures offer a direct window into the perceptual processes underlying action recognition and highlight the influence of expertise upon them.

**Figure 1:**
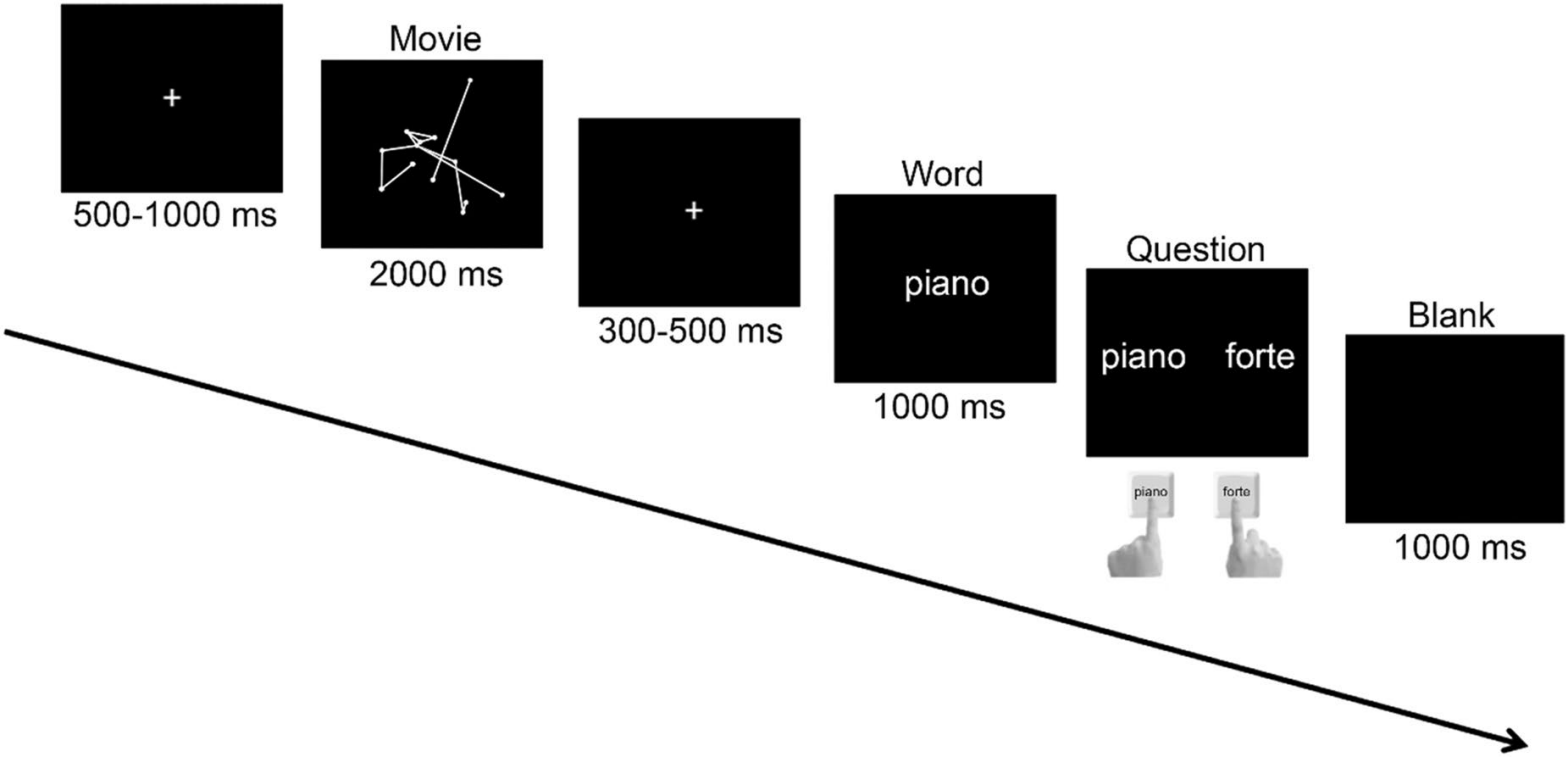
Overview of the displays and timing of events in the experiment. A movie displaying a violinist playing either a forte or piano musical nuance was presented before the word forte or piano, then participants had to tell if the violinist in the movie played a forte or piano musical nuance with the left and right index fingers.

The aim of the present study was to investigate how semantic congruency and incongruency could interfere with musical gesture decoding, and to what extent such interference would depend on the level of expertise (i.e. of automaticity developed by the participant). To answer this question, we used an original backward priming paradigm (Fig. 1) which combines gesture and linguistic components. Specifically, a semantically related or unrelated word followed a muted video of a violinist expressing a *piano* (i.e. a slow and soft movement) or *forte* (i.e. a rapid and strong movement) musical nuance that musically trained (with an average of 10.08 years of music practice, see “Methods” section) and untrained (with an average of 0.70 years of music practice, see “Methods” section) participants were asked to categorize. The influence of the word on the musical gesture decision was investigated through the N400 event-related potentials. N400 is a negative deflection occurring 200 to 600 ms after stimulus onset reflecting incongruency detection in the context of a preceding stimulus^26^. A second component attracted our attention around 200 ms, whose topography appears to correspond to the P200 component. Generally evoked between around 150 to 250 ms after the onset of visual or auditory stimuli, the P200 component is consensually thought among researchers to be involved in higher-order perceptual processing, modulated by attention^27–29^. The relation between the P200 and N400 component was thus further investigated in our study. We hypothesized that musically trained participants would be more efficient at recognizing the musical nuances expressed by the violinist due to their knowledge and integration of music-related motor experience. They should be faster and more accurate than untrained participant at making their judgements. As we predict a strong language-action interaction, both groups should be faster and more accurate with semantically congruent than incongruent words. However, based on their knowledge, and integrated motor experience of the musical nuances, trained participants should be more confident in their decision and less influenced by the word displayed after the video. Therefore, the difference between the congruent and incongruent conditions should be stronger for the untrained group than for the trained group. At the electrophysiological level, both groups should reveal an incongruency detection during word presentation as reflected by the N400 component, but this activation should be more important for untrained participants.

## Results

### Preprocessing

Trials with incorrect (12.83% of total trials), missing (> 3 standard deviations above the mean; 1.90% of total trials), or anticipatory (< 3 standard deviations under the mean; 0% of total trials) responses were excluded from the data.

### Behavioural results

To test our behavioral assumptions, we used the generalized linear mixed models (GLMMs) statistical method. An advantage of GLMMs compared to more classical analyses of variance is the possibility to add random effects in our statistical models, like inter-indivual performances^30,31^. Moreover, GLMMs handle non-Gaussian data by allowing the specification of various distributions, such as a binomial distribution for the accuracy analysis, modeling the total variance of our data including the inter-trial variance^30^.

**Figure 2:**
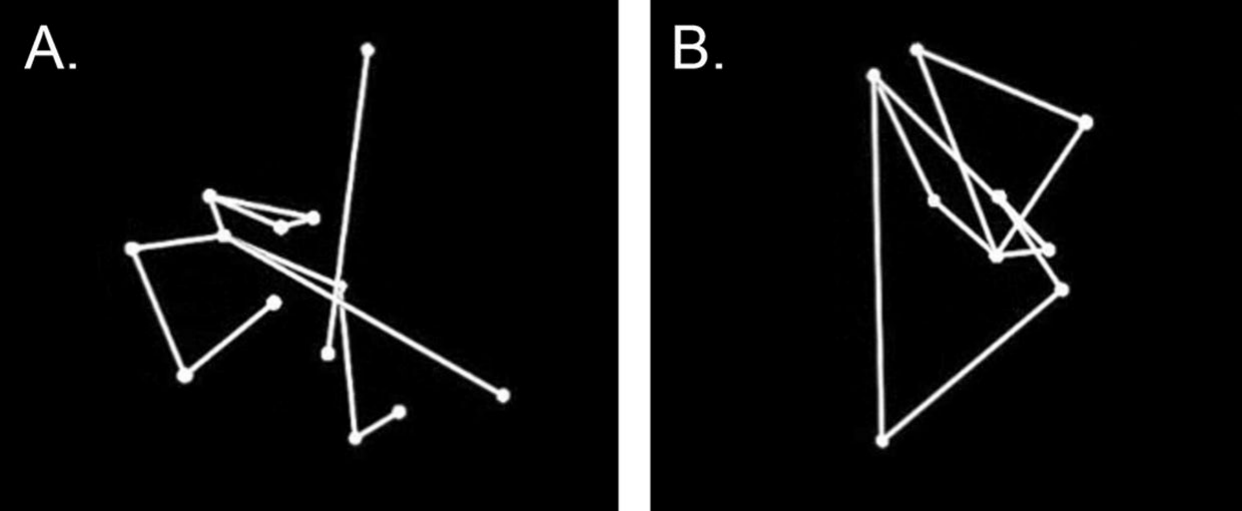
Two examples of stimuli presented during the experiment. **(A)** Stimulus normally displayed and **(B)** scrambled version of the stimulus.

Our dependent variables were the reaction time and accuracy to decide about the musical nuance played by the violinist. To investigate the contribution of each variable and their interaction, we compared different models using the Fisher’s F test for the reaction time, and the chi-square difference test specifying binomial models for the accuracy. Our fixed effects were the congruency (congruent vs. incongruent) and instrumental practice (trained vs. untrained). To focus participants’ attention on the violinists’ gestures and reduce potential effects due to their appearance, violinists’ performance was only represented by 13 point-lights attached to their head and major articulation points (neck, shoulders, elbows and wrists), as well as to their violin and bow (see Fig. 2; for a sample video of the stimuli see Video A and Video B of the supplementary materials section). The latter control was increased by adding another fixed variable: The video display (normal vs. scrambled). In the scrambled condition, videos displayed scrambled versions of violinists’ performance, created by randomizing the starting positions of the point-lights. In the normal condition, videos were presented as captured. Our random effects were the inter-individual performance and violinists’ identity.

#### Reaction time

The only significant effect in the reaction time analysis was the video display main effect, *F*(1, 3,800) = 15.01, *p* < 0.001, $R_{m}^{2}$  < 0.01, $R_{c}^{2}$ = 0.33 (Note: $R_{m}^{2}$ representss the variance explained by the fixed factors, while $R_{c}^{2}$ is the variance explained by both fixed and random effects^32^). Participants were faster to judge the nuance expressed by the violinist when the video was normally displayed compared to the scrambled condition. The congruency, *F*(1, 3,800) = 0.76, *p* = 0.38, and instrumental practice, *F*(1, 18) = 0.14, *p* = 0.71, main effects, as well as the interaction between them, *F*(1, 3,800) = 0.36, *p* = 0.55, were not significant for the reaction time. Moreover, neither the interaction between the video display and congruency, *F*(1, 3,800) = 0.18, *p* = 0.67, the video display and instrumental practice, *F*(1, 3,800) = 1.64, *p* = 0.20, nor the three-way interaction between the video display, congruency and instrumental practice, *F*(1, 3,800) = 0.17, *p* = 0.68, were significant.

#### Accuracy

**Figure 3:**
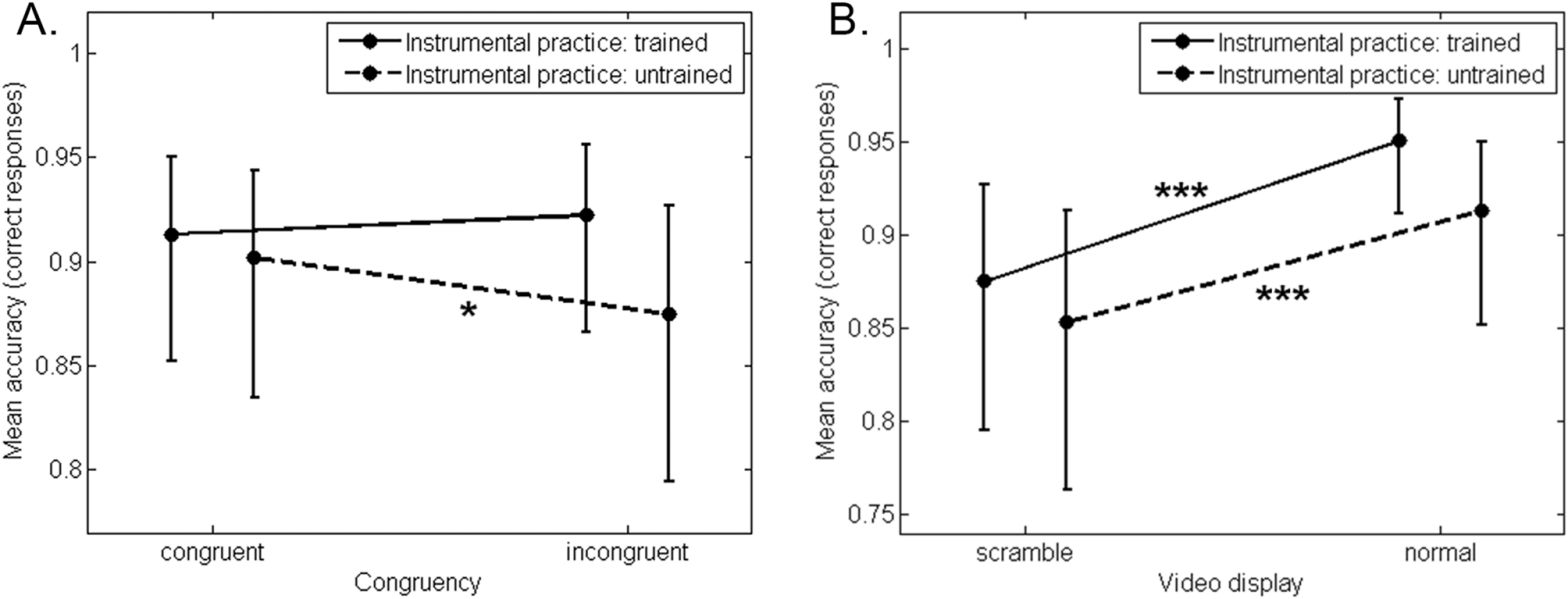
Accuracy results. **(A)** Interaction between the congruency and instrumental practice factor. **(B)** Interaction between the video display and instrumental practice factor. *** corresponds to a significance level of .001 and * to a significance level of .05. Vertical bars represent confidence intervals of .95.

Although, neither the congruency, χ^2^(1, *N* = 20) = 1.10, *p* = 0.29, nor the instrumental practice, χ^2^(1, *N* = 20) = 0.84, *p* = 0.36, factors showed a significant main effect, the two-way interaction between these variables was significant, χ^2^(1, *N* = 20) = 4.15, *p* < 0.05, $R_{m}^{2}$ < 0.01, $R_{c}^{2}$ = 0.19 (Fig. 3A). As shown by simple effects, untrained participants were more accurate in the congruent condition than in the incongruent condition, χ^2^(1, *N* = 20) = 4.58, *p* < 0.05. The same effect was not significant for trained participants, χ^2^(1, *N* = 20) = 0.70, *p* = 0.40. Interestingly, the video display main effect was significant, χ^2^(1, *N* = 20) = 62.66, *p* < 0.001, $R_{m}^{2}$ = 0.03, $R_{c}^{2}$ = 0.22, as well as its interaction with the instrumental practice, χ^2^(1, *N* = 20) = 4.30, *p* < 0.05, $R_{m}^{2}$ = 0.05, $R_{c}^{2}$ = 0.23 (Fig. 3B). Concerning the main effect, participants were more accurate to judge the nuance expressed by the violinist when preceded by a normal than scrambled video. For the interaction, both trained and untrained participants were more accurate in the normal than scrambled condition, χ^2^(1, *N* = 20) = 42.61, *p* < 0.001 and χ^2^(1, *N* = 20) = 21.17, *p* < 0.001 respectively, but the normal-scrambled difference was significantly more important for trained than untrained participants, χ^2^(1, *N* = 20) = 4.48, *p* < 0.05. Neither the interaction between the video display and congruency, χ^2^(1, *N* = 20) = 0.50, *p* = 0.48, nor the three-way interaction between the video display, congruency and instrumental practice, χ^2^(1, *N* = 20) = 0.73, *p* = 0.39, were significant.

To ensure that the differences observed between trained and untrained participants were not due to differences in strategy (or response biases) between groups, we calculated the response bias “c” index. The c index is a statistic used in signal detection theory^33^, representing response biases, by taking into account both “hits” (correctly detecting the nuance when the video and word are congruent) and “false alarms” (respond according to the word when it is incongruent with the video) in the calculation of task performance. The c index was calculated for the normal videos in combination with the scrambled ones. Comparison of the c parameter for trained (*M* = − 0.10; *SD* = 0.13) and untrained (*M* = − 0.05; *SD* = 0.18) participants, using a Student independent sample t-test, revealed no significant difference between the groups, *t*(21) = 0.66, *p* = 0.52, *d* = 0.32.

### ERP results

**Figure 4:**
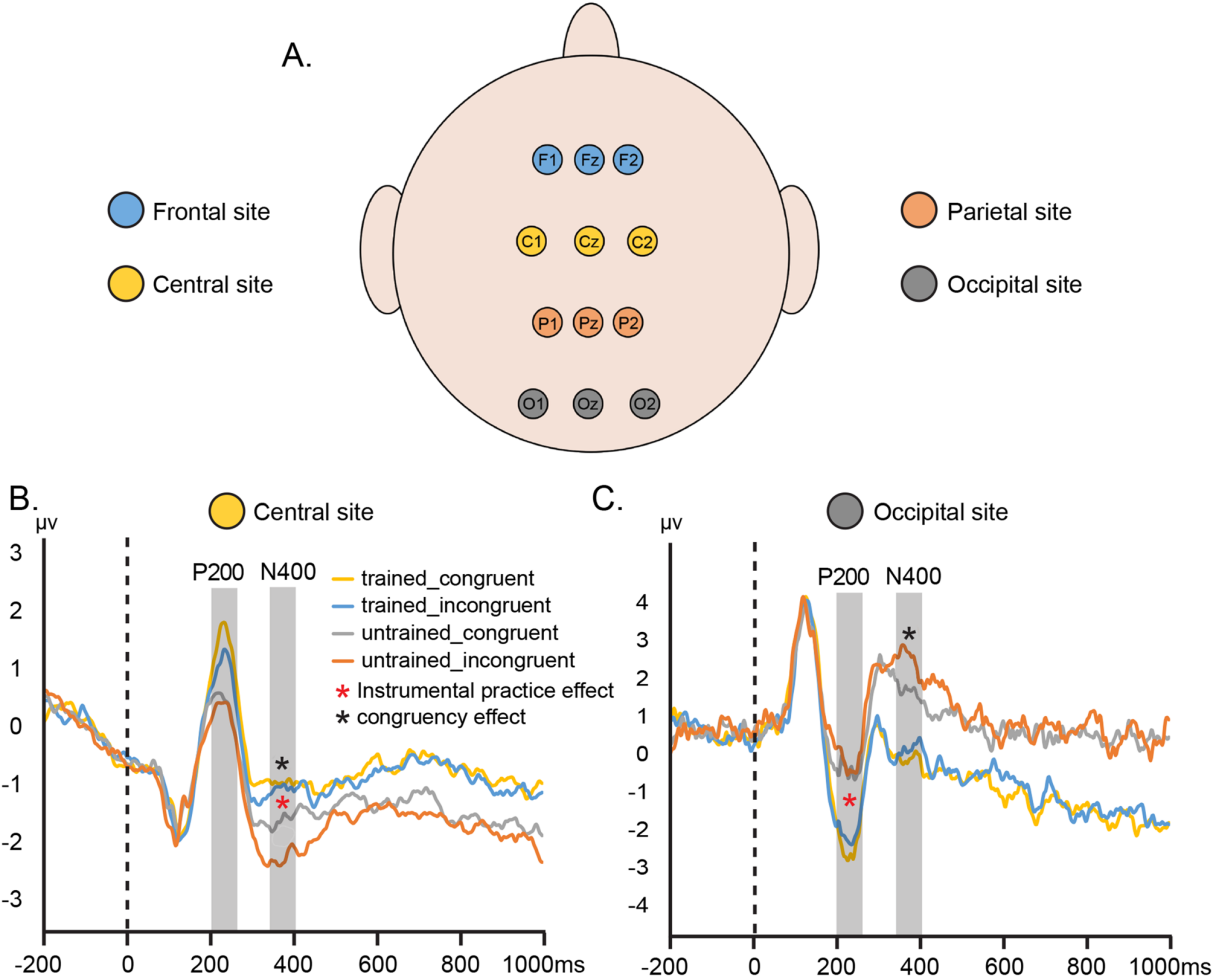
**(A)** Schematic illustration of the electrode grouping method used to analyze data in the experiment. **(B)** Event-related potential result in mean amplitude µv for the central site (C1, Cz and C2). **(C)** Event-related potential result in mean amplitude µv for the occipital site (O1, Oz and O2).

We focused our ERP analyses on the 200 ms before and 1,000 ms after the presentation of the word *forte* and *piano*. Average ERPs were computed in response to the congruency and instrumental practice conditions and the mean amplitude in a 3-electrode site array, divided in 4 zones (Fig. 4A): frontal (F1, Fz, F2), central (C1, Cz, C2), parietal (P1, Pz, P2) and occipital (O1, Oz, O2). We chose to gather these electrodes based on previous studies investigating the N400 component, showing that its effect was maximal at centro-parietal electrode sites^34,35^.

A mixed ANOVA was conducted on the mean amplitude of the ERPs over 350 to 400 ms after stimulus onset for the N400, with the factor congruency (congruent vs. incongruent, within-participants) × instrumental practice (trained vs. untrained, between-participants) × electrode site (frontal vs. central vs. parietal vs. occipital, within-participants) × video display (normal vs. scrambled, within-participants). 210 to 260 ms after stimulus onset, a deflection possibly reflecting the P200 component seemed to differentiate our trained participants to our untrained participants. To statistically test this component, we conducted another mixed ANOVA with the same factors on this time window. Moreover, in order to test the relation between the P200 and N400 event related potentials we performed a Pearson correlation analysis on the P200 at occipital site and the N400 at central site independently of the congruency and video display conditions. The dependent variable was the mean amplitude.

### N400

A summary of N400 results can be found in Table 1. We found a significant main effect of the electrode site. A contrast analysis showed that N400 mean amplitude was significantly more negative in the central electrode site than in other electrode sites, *t*(18) = − 4.56, *p* < 0.001. N400 mean amplitude was significantly more positive in the occipital electrode site than in other electrode sites, *t*(18) = 2.15, *p* < 0.05. N400 mean amplitude was not significantly different in frontal and parietal electrode sites, *t*(18) = 0.03, *p* = 0.98. Neither the main effects of congruency nor instrumental practice were significant. However, the video display showed a significant main effect in which N400 was more negative when the word was preceded by a normal than scrambled video.

**Table 1:** Summary of N400 results.

Effect	*F*(*df*)	*p*	$\eta_{p}^{2}$
Electrode site (1)	*F*(3,54) = 3.95	< 0.05	0.18
Musical practice (2)	*F*(1,18) = 0.06	0.94	< 0.001
Congruency (3)	*F*(1,18) = 0.10	0.75	0.01
Video display (4)	*F*(1,18) = 18.81	< 0.001	0.51
1 × 2	*F*(3,54) = 2.88	< 0.05	0.14
1 × 3	*F*(3,54) = 4.22	< 0.01	0.19
1 × 4	*F*(3,54) = 0.92	0.44	0.05
2 × 3	*F*(1,18) = 0.58	0.47	0.03
2 × 4	*F*(1,18) = 0.35	0.56	0.02
3 × 4	*F*(1,18) = 0.17	0.69	0.01
1 × 2 × 3	*F*(3,54) = 1.69	0.18	0.09
1 × 2 × 4	*F*(3,54) = 0.18	0.91	0.01
1 × 3 × 4	*F*(3.54) = 1.45	0.24	0.07
2 × 3 × 4	*F*(1,18) = 2.56	0.13	0.12
1 × 2 × 3 × 4	*F*(3,54) = 0.09	0.96	0.01

Concerning interactions, two significant effects were obtained for N400: An interaction between the congruency and electrode site, and between the instrumental practice and electrode site. Concerning the congruency-electrode site interaction, simple effects showed that in the central electrode site, the mean amplitude of N400 was more negative for the incongruent condition, *t*(18) = 2.35, *p* < 0.05 (Fig. 4B). In the occipital electrode site, the mean amplitude of N400 was more negative for the congruent condition, *t*(18) = − 2.19, *p* < 0.05 (Fig. 4C). No significant differences between the two congruency conditions for frontal, *t*(18) = 1.74, *p* = 0.10, and parietal, *t*(18) = 0.02, *p* = 1, electrode sites were found (Figure A of the supplementary materials section).

For the instrumental practice-electrode site interaction, N400 mean amplitude was more negative in the central electrode site for untrained than trained participants, *t*(18) = − 2.43, *p* < 0.05 (Fig. 4C). No significant differences between trained and untrained participants were found for the frontal, *t*(18) = − 1.30, *p* = 0.21, parietal, *t*(18) = 0.10, *p* = 0.92, and occipital, *t*(18) = 1.88, *p* = 0.08, electrode sites.

### P200

A summary of P200 results can be found in Table 2. Only one significant main effect was significant for P200: The electrode site. A contrast analysis showed that the early component mean amplitude was significantly more negative in the occipital electrode site than the other electrode sites, *t*(18) = − 4.97, *p* < 0.001.

**Table 2:** Summary of P200 results.

Effect	*F*(*df*)	*P*	$\eta_{p}^{2}$
Electrode site (1)	*F*(3,54) = 15.13	< 0.001	0.46
Musical practice (2)	*F*(1,18) = 0.01	0.92	< 0.001
Congruency (3)	*F*(1,18) = 1.58	0.23	0.08
Video display (4)	*F*(1,18) = 3.62	0.07	0.17
1 × 2	*F*(3,54) = 3.65	< 0.05	0.17
1 × 3	*F*(3,54) = 1.66	0.19	0.08
1 × 4	*F*(3,54) = 1.79	0.16	0.09
2 × 3	*F*(1,18) = 0.58	0.46	0.03
2 × 4	*F*(1,18) = 0.72	0.41	0.04
3 × 4	*F*(1,18) = 0.02	0.90	< 0.001
1 × 2 × 3	*F*(3,54) = 0.86	0.47	0.05
1 × 2 × 4	*F*(3,54) = 0.44	0.72	0.02
1 × 3 × 4	*F*(3,54) = 0.60	0.62	0.03
2 × 3 × 4	*F*(1,18) = 0	0.97	< 0.001
1 × 2 × 3 × 4	*F*(3,54) = 0.06	0.98	< 0.01

The only significant interaction observed for P200 was the one between the electrode site and instrumental practice. Simple effects showed that the early component mean amplitude was significantly more negative in the occipital electrode site for trained than untrained participants, *t*(18) = 2.14, *p* < 0.05 (Fig. 4B). There was no significant differences between the instrumental practice groups for frontal, *t*(18) = − 1.63, *p* = 0.12, central, *t*(18) = − 1.80, *p* = 0.09, and parietal, *t*(18) = 0.24, *p* = 0.82, electrode sites (Figure A of the supplementary materials section).

### P200 vs. N400 correlation

The Pearson correlation analysis comparing the relation between the amplitude of the P200 at occipital site and the amplitude of the N400 at central site revealed a significant effect, *r*(18) = -0.66, *p* < 0.01 (Figure B of the supplementary materials section).

## Discussion

Both trained and untrained participants successfully recognized the musical nuances expressed by the violinist’s muted gestures (87% of correct responses in both populations). Taken separately, the congruency and instrumental practice effects were not significant regarding reaction time and accuracy analysis of the recognition task. Contrary to what was expected, trained participants were not significantly better than untrained participants at recognizing the musical nuances. However, the congruency-instrumental practice interaction revealed significant differences between the instrumental practice groups in the accuracy analysis. Untrained participants were more accurate when the word and gesture were semantically related in the congruent condition, with respect to the incongruent condition. No such significant difference was observed in the musically trained group, who showed relatively stable performances between both conditions. These observations suggest that the word had an influence in the gesture decoding of the untrained group only. However, further electrophysiological results nuance this conclusion.

Although only untrained participants were behaviourally influenced by the presentation of the word (i.e. in the accuracy analysis), a significant N400 component was observed regardless of the instrumental practice group. The mean amplitude of the ERP was significantly greater for the incongruent condition at central and occipital (reversed effect due to the polarity^35^) site, indicating that the mismatching relation between word and gesture was due to the processing of semantic information. Supporting Koelsch et al.^24^, the present results indicate that language and music share the ability of transferring semantic information. In fact, our study went further showing that this effect can be independent of musical practice level and that even muted musicians’ gestures are able to express meaningful information. Interestingly, the amplitude of the N400 at the central site was greater for untrained than for trained participants, independently of the congruency-incongruency difference, whereas no differences between groups were found for the corresponding N400 at occipital site (of note: N400 effect is maximal at centro-parietal electrode sites^34,35^). The greater N400 activation at the central site in untrained participants, corroborated by their behavioral results showing significant differences between the congruent and the incongruent condition, tends to show that untrained participants were more likely to rely on word-related information to perform the task. Referring to the action-perception coupling theory^2^ and the internal forward model^5^, a possible explanation would be that as untrained participants have not encoded the sensorimotor properties of *piano* and *forte* musical movements through musical practice (i.e. they do not have any representation of such concept at sensorimotor level, because they never practiced such movement), they would then refer more to external cues (i.e. the word) to make a decision compared to trained participants. This explanation is in accordance with the results related to the scrambled display, where the anthropomorphic organization of body limbs are disrupted. Although both trained and untrained participants seemed to benefit from the normal video display condition (i.e. faster reaction times and better accuracy), the significant interaction between the video display and instrumental practice in the accuracy analysis reveals that the difference between the normal and scrambled display is greater for trained than for untrained participants. It seems that a human-body like shape may reinforce the perceptual decision-making process, especially for people with integrated motor experience (i.e. trained participants). Results regarding N400 activations revealed a greater N400 for the normal video display condition in both groups, indicating that incongruency effects are strengthened when a human shape is displayed.

Observing in further detail our electrophysiological results, we found another difference between our groups of participants. From 210 to 260 ms at occipital site, trained participants revealed a greater P200 component than untrained participants. Consensually considered by researchers as representing higher-order perceptual processing, modulated by attention, P200 could reflect a necessary step in the orienting response towards relevant stimuli, to allow for the subsequent allocation of attention resources by the posterior brain regions responsible for the completion and integration of sensory information^27–29^. Literature also suggests that P200 is able to distinguish between congruent and incongruent targets in a priming task and could thus be implicated in the congruency detection at a more perceptual level than the N400^27^. However, in the present experiment, only the expertise effect was significant for the P200 at occipital site, not the congruency effect. Maybe that the difference observed between trained and untrained participants at P200 in the occipital area could be interpreted as a higher overall level of activation in the occipital region for trained than for untrained participants. Indeed, as noticed by a reviewer, it may be that trained participants are more involved in analyzing the visual information contained in the video thereby possibly increasing both visual perception and visual attention. However, we note that contrary to the P200, the N400 component did not significantly distinguish trained and untrained participants at the occipital level, contradicting the idea of a global greater occipital activation in untrained participants.To better understand the relationship between the P200 obtained at occipital site and the previously observed N400 at central site, we performed a Pearson correlation analysis between these two components and found that they were negatively correlated: The greater P200’s amplitude, the smaller N400’s amplitude. It seems that trained participants spent more resources than untrained participants to process the word at P200, which further reduced the amplitude of their N400 component. Given the occipital location of the component and as the congruency effect is not significant at the level of P200, the most parsimonious interpretation of the greater activation of this potential in trained participants is that this group activated more low-level processes, most probably visual perception and/or visual integration, than untrained participants when presented with the word to integrate the information previously processed from the video. Although the incongruency between the musical nuance and the word was detected regardless of the instrumental practice, trained and untrained participants differed not only in their use of the word, but also in the electrophysiological processing of the word. Due to their motor and semantic expertise of the musical field, trained participants might be able to construct more elaborated percept of the word than untrained participants. These results could be interpreted in terms of different strategies used by trained and untrained participants. Trained participants would activate lower level mechanisms, at early stage of processing, linked to visual perception and/or integration, acquired through extensive training and practice, which as a consequence reduce their semantic processing at N400. Untrained participants would beneficiate less from low-level processing and rather activate more semantic processes at N400. An alternative interpretation would be that the knowledge of musical gestures acquired by trained participants makes this group faster at recognizing the musical nuance expressed by the violinists. Our participants had up to 2,500 ms before the word appeared on the screen (i.e. the time of the movie and the following fixation cross). Thus, trained participants might activate more visual perception/integration, highlighted by their greater P200 potential, when the word is presented, because they have already made their gesture decision and they are no longer allocating processing resources to this aspect of the task. Following this idea, only untrained participants would be more inclined to follow word-gesture semantic association in their behavioural performance, because they are still indecisive. Both interpretations are plausible and highlight the ability of musicians to decode what they perceive once they recognize human-form or movements related to music^36^. Whether or not trained participants have already made their decision about the gesture expressed, the P200 reflects an earlier processing of the word in this group, which has the effect of reducing the impact of the subsequent N400. Both groups significantly react to the word-gesture ingruency at N400, but the amplitude of this component is reduced in trained participants, because resources were already involved in P200 at a more perceptual level.

### Limitations and future directions

Our study did not show any significant differences on the reaction time analysis, whereas studies using a priming task in the literature mainly consider it as an indicator of an interference effect^20,23,37^. Bernardis and Caramelli^20^, for instance, found that when words and iconic gestures were incongruently associated, participants' mean reaction time was significantly greater. In our study, the stimuli used were not iconic but musical gestures. Participants were also not asked to directly describe what they saw (e.g., a violinist playing music), but asked to define the musical nuances expressed by the gesture (i.e. *piano* or *forte*). Whereas iconic gestures are easily recognizable by everyone, musical gestures convey several information more accessible with musical practice (i.e. the more an action is practiced, the better is it recognized and anticipated^2^). When confronted with musical gestures, one needs to consider their functional aspects (e.g., the speed and trajectory), as well as the expressive and affective aspects (i.e. the emotional information given by the body movements of the musicians^38^). Musical nuances expressed by these muted gestures are not directly recognizable at a glance and require collecting all this panel of information to be interpreted. Musically trained participants learnt this information (i.e. functional, affective, and expressive) through extensive musical training. Therefore, musicians observing musical gestures may be slowed by the amount of information they can reach from them compared to non-musicians (e.g., musicians will analyze a slow gesture more thoroughly than non-musicians, who will consider it simply as slow equal *piano*), erasing cross-group differences in reaction time.

Another discrepancy with the literature was found in our study, concerning this time the P200 and N400 effects at the level of the parietal site. Indeed, although not all studies systematically highlight their results at the level of the parietal site, when they do, the P200 and N400 components are generally visible in this area^34,35^. We cannot tell with certitude why this is not the case in our study. Data pre-processing has been done with care and no systematic technical problems have been observed with our parietal electrodes of interest during recording. The P200 and N400 components are generally maximally observed at centro-parietal electrodes^26^. We note, that results of our study do not contradict this conclusion as the P200 and N400 effect are visible at the frontal, central, centro-parietal (not reported) and occipital electrodes, with a maximum effect around central electrode site.

Our study clearly demonstrates an interrelation between language, music and gestures in the light of our behavioral (i.e. accuracy analysis) and electrophysiological results. Corroborating previous literature about gesture and speech^20^, as well as music and speech^24^, our study goes further revealing differences in the processing of a word semantically related or not to a previous musical gesture, as well as a different use of linguistic information to perform the task depending on the level of expertise. Using muted-musical gestures instead of musical sounds makes our study stand out from previous research in the domain as it provides new insights about the perception of music-related information in muted environment and offers a good framework for non-verbal behavioral study. Future studies could improve our experimental setup by using words evoking intermediate emotions instead of the clear distinction between ‘forte’ and ‘piano’ words. Whereas distinct use of the word depending on the musical expertise was highlighted in the present experiment, our results probably minimized the scope of these differences. By only presenting 2 words, both groups may have favored from a habituation effect which could particularly help untrained participants, reducing differences between groups. Another adjustment could sensitively potentiate expertise effects:Ask professional musicians instead of trained participants to perform the task. Professionals, who cumulate many years of practice, would probably enhance the effect with even more automated processes. Indeed, according to the action-perception coupling view, motor expertise is essential to predict and understand an action^2^, and professional violinists should then amplify the contrast with untrained participants.

## Conclusion

The aim of the present study was to investigate how language is involved in perception–action relations and the role of expertise in this interaction. Using an original backward priming paradigm, we revealed that an incongruent word was able to interfere with musical gesture detection regardless of the musical practice. Our results also revealed differences in processes of this word at electrophysiological level between trained and untrained participants. Whereas musically trained participants tended to activate lower level processes, at early stage of processing, probably related to visual perception and/or visual integration as reflected by a greater P200 potential at occipital site, musically untrained participants activated higher cognitive processes as reflected by the N400 component at central site. The P200 at occipital site and the N400 component at central site were negatively correlated regardless of the musical practice group, which means that the greater one component was, the smaller the other. Interestingly, trained and untrained participants not only differed in their electrophysiological activation but also in their behavioral performance. Untrained participants were more inclined at following word-gesture semantic association in their decision concerning the musical nuance, while trained participants, benefiting from their expertise, were more decisive and referred to their own evaluation.

Together, our study was able to improve knowledge on the relation between action, perception and language at the electrophysiological and behavioral level, revealing musical expertise effects and the major role of semantic knowledge in visual decision-making of music expressivity, making our results stand out from previous research in the domain.

## Methods

### Participants

Twenty naive volunteers participated to this study for course credits or remuneration. The sample size was decided based on a power analysis realized on 6 pilot participants (3 musically trained and 3 musically untrained participants) with the “simr” package on R^39^. The musically trained pilot participants practiced music on average 8.33 years (*SD* = 0.58), and the untrained pilot participants on average 0.33 years (*SD* = 1.53). 85.80% of power (95% CI, [83.48, 87.91]) for an effect size of $R_{m}^{2}$ = 0.004 and $R_{c}^{2}$ = 0.27 was obtained by simulating a sample size of 20 participants.

The 20 participants of the experiment were students from the University of Geneva recruited based on their level of musical practice measured by an in-house questionnaire. Untrained participants (*N* = 10; 4 males; *M*_*age*_ = 22.2 years, *SD* = 2.2) practiced music between 0 and 2 years (*M* = 0.70 years; *SD* = 0.90) and did not currently practice music. Trained participants *(N* = 10; 1 male; *M*_*age*_ = 22.44 years, *SD* = 1.26) practiced music from 5 to 14 years (*M* = 10.08 years; *SD* = 2.67). More details on the musical practice of the participants can be found in Table A of the supplementary materials section. All participants had normal or corrected-to-normal vision and no neurological or psychiatric conditions. The study was approved by the local ethical comity of the University of Geneva and written informed consent was obtained from participants before the experiment. Moreover, all methods were carried out in accordance with relevant guidelines and regulations as stated in the Declaration of Helsinki.

### Materials and procedure

Participants were equipped with an electrocap to record electroencephalography (EEG) and comfortably installed in a Faraday cage in front of a Philips 242G5D 24″ monitor, attached to a Dell OptiPlex 9,020 Intel Inside Core i7 vPro computer. They were invited to perform the following task while recording EEG (Fig. 1): A fixation cross was presented for 500 to 1,000 ms before the video of a violinist playing a forte or piano musical nuance for 2000 ms. A second fixation cross appeared then for 300 to 500 ms, followed by the word *forte* or *piano* displayed in the center of the screen for 1,000 ms. No specific instruction was given for the word. In total, 224 muted videos composed of a factorial combination of 7 violinists × 2 musical nuances (forte vs. piano) × 2 video displays (normal vs. scramble) × 2 congruency conditions between the word and the gesture (congruent vs incongruent), repeated four times in order to have enough trials for the EEG analysis, were presented to the participants. Half of trials were congruent and the other half were incongruent. The choice of putting the stimulus to interpret, here the video, before the interferent stimulus, here the word, whereas the opposite is generally observed in classical priming paradigms^19,20^, was to put the emphasize on the gesture analysis. Participants were focused on the decision whether the violinist was playing a piano or forte nuance and the word was only a secondary information that could be used in the event of indecision. In such backward priming paradigm, showing a semantic interference of the word in the decision concerning the musical nuance expressed by the violinist, should emphasize a strong link between action and language. Immediately after the word, participants had to decide if the gesture played by the violinist was a forte or piano musical nuance by pressing the left and right arrow of a standard keyboard with their respective index fingers. Half of participants used the left arrow to respond to a *piano* musical nuance and the right arrow to a *forte* musical nuance. The other half had opposite instructions. Finally, a black screen was displayed for 1,000 ms before the next trial. The experiment lasted approximately 1 h and was divided in 8 blocks of 28 trials allowing participants to rest between blocks.

### EEG acquisition and raw data processing

The same EEG methodology as the one of a previous experiment of our group was used in this study^40^. The EEG was recorded using the "Biosemi" system (Amsterdam, Netherlands). This apparatus includes an ActiveTwo amplifier system AD-Box with 64 active AG/AgCL electrodes. The electrodes were sampled at 1,024 Hz in a bandwidth filter of 0 to 268 Hz. In addition, we detected the saccades and blinks using an electro-oculogram. In order to do so, we measured the voltage difference between two horizontal electrodes (HEOG) located at the outer canthus of each eye, and two vertical electrodes (VEOG) above and below the right eye. An averaged reference (average potential subtracted from the value of each recording channel) was used as *online* and *offline* references.

The Brain Vision Analyzer 2.2 software (Brain Products, Gilching Germany) was used to perform raw data pre-processing. Data were band-pass filtered offline between 0.15 and 70 Hz within a Butterworth zero phase filter. In addition, a 50 Hz notch removed noise from the power-line. Finally, data were down-sampled to 500 Hz in order to reduce data size and thus processing time. A baseline correction of 200 ms before stimulus onset was performed before excluding artifacts. Through independent component analysis, ocular artifacts were identified and corrected. Artifact rejection was applied to remove voltages under − 100 μv and above 100 μv. On average 3.33% (*SD* = 3.83) of the trials per experimental condition were removed. Finally, noisy channels were interpolated by means of a 3-D spherical spline. On average 2.73% of the electrodes were corrected this way (*SD* = 1.48).

## Supplementary information

Supplementary Information.

Supplementary Video 1.

Supplementary Video 2.

## Data Availability

Under the Swiss guidelines of data protection (Ordinance HFV Art. 5), the datasets generated and analyzed during the current study can be made available from the corresponding author on a case by case basis.
