# Supplementary material for: Language is the missing link in action-perception coupling: an EEG study
Source: Sci Rep. 2020 Sep 3;10:14587. doi: 10.1038/s41598-020-71575-w (PMC7471270; doi:10.1038/s41598-020-71575-w)
Supplement: Supplementary file 1 — Supplementary Information. [file 41598_2020_71575_MOESM1_ESM.pdf]

Supplementary materials

Language Is the Missing Link in Action-Perception Coupling: An EEG Study

Pauline Billard[1,+], Sélim Yahia Coll[1,*,+], Donald Glowinski[1,++] and Didier Grandjean[1,++]

[1]'Neuroscience of Emotion and Affective Dynamics' laboratory, Faculty of Psychology and Educational Sciences and Swiss Centre for Affective Sciences, University of Geneva, Geneva, 1205, Switzerland

*coll.selim@gmail.com

[+]First co-authors (equal contribution)

[++]Senior co-authors (equal contribution)

Video A. Example of "scrambled" stimulus presented during the experiment. The video depicts a violinist playing a forte nuance.

Video B. Example of "normal" stimulus presented during the experiment. The video depicts a violinist playing a piano nuance.

	Untrained (*N* = 10)	Trained (*N* = 10)
Instrumental practice	Yes: 4	Yes: 10
	No: 6	No: 0
Main instrument played	None: 6	None: 0
	Piano: 3	Piano: 7
	Trumpet: 0	Trumpet: 1
	Guitar: 0	Guitar: 2
	Violin: 1	Violin: 0
Practice of second instruments	Yes: 0	Yes: 4
	No: 10	No: 6
Nature of second instruments		Fifre: 1
		Piano: 2
		Unknown: 1[1]
Current musical practice	Yes: 0	Yes: 3
	No: 10	No: 7
Subjective instrumental level	None: 6	None: 0
	Beginner: 4	Beginner: 0
	Intermediary: 0	Intermediary: 7
	Advanced: 0	Advanced: 3
Music degree	Yes: 0	Yes: 3
	No: 10	No: 7

Table A. Details regarding the musical practice of trained and untrained participants. Numbers correspond to the distribution of trained and untrained participants in each category.

[1]The participant did not provide this information

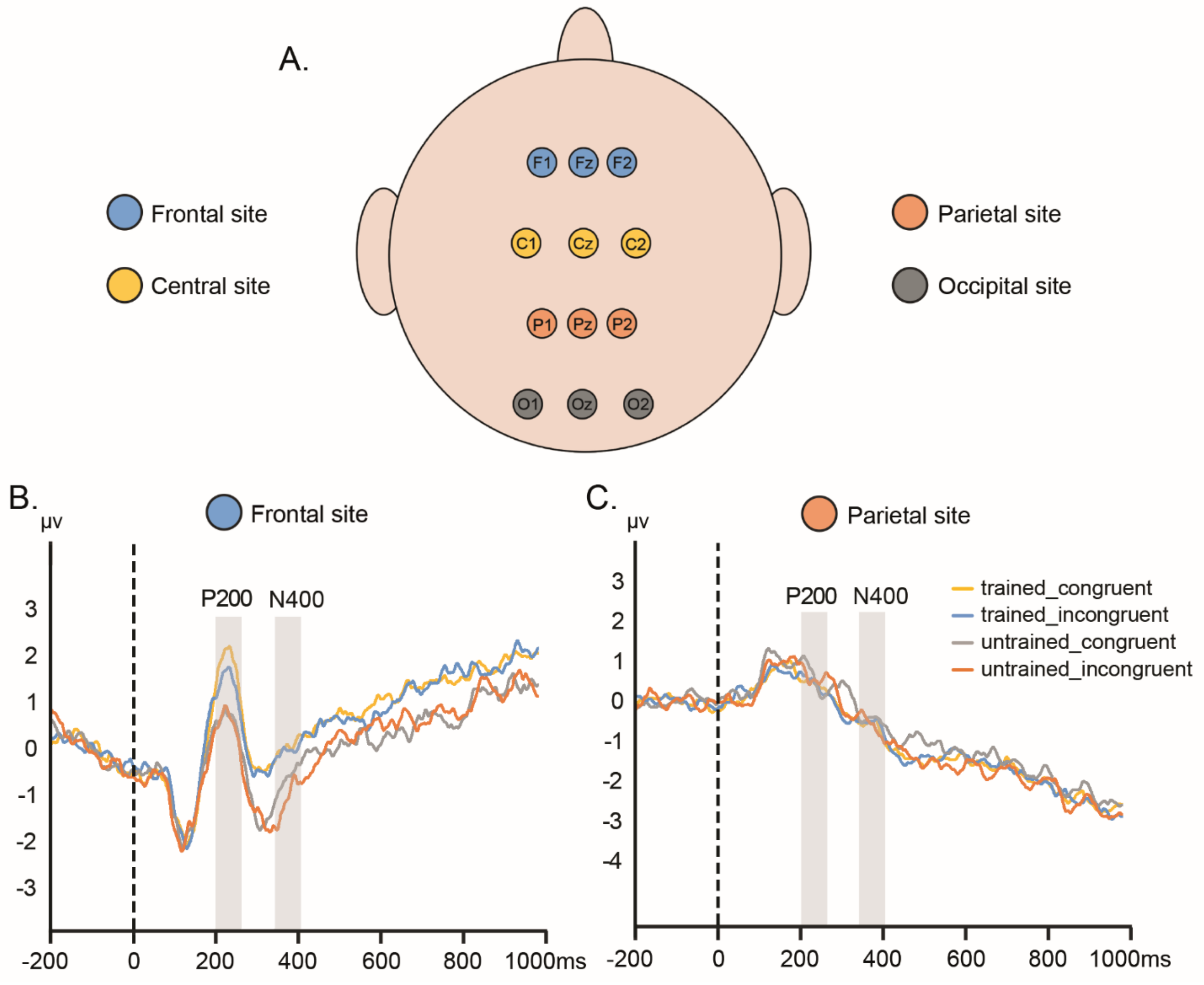

Figure A. A. Schematic illustration of the electrode grouping method used to analyze data in the experiment. B. Event-related potential result in mean amplitude μv for the frontal site (F1, Fz and F2). C. Event-related potential result in mean amplitude μv for the parietal site (P1, Pz and P2).

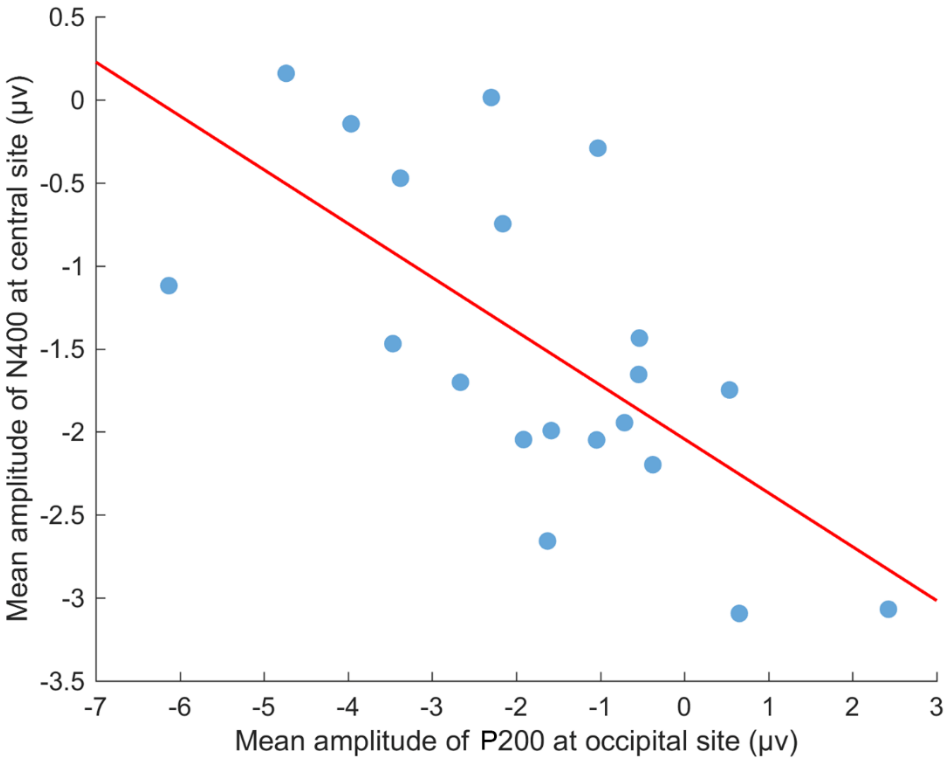

Figure B. Scatter plot of the correlation between the mean amplitude of P200 at occipital site and the mean amplitude of N400 at central site independently of the congruency and video display conditions. The regression line is represented in red.
